# Panniculitis as an Initial Presentation of Dermatomyositis: A Case Report

**DOI:** 10.31729/jnma.4827

**Published:** 2020-03-31

**Authors:** Anjal Bisht, Niraj Parajuli, Monasha Vaidya, Sumida Tiwari

**Affiliations:** 1 Department of Medicine, National Academy of Medical Sciences, Kathmandu, Nepal; 2 Department of Dermatology, National Academy of Medical Sciences, Kathmandu, Nepal; 3 Department of Pathology, Kantipur Dental College and General Hospital, Basundhara, Nepal; 4 Department of Pathology, National Academy of Medical Sciences, Kathmandu, Nepal

**Keywords:** *dermatomyositis*, *erythema*, *myopathy*, *panniculitis*

## Abstract

Dermatomyositis is an idiopathic muscle disease characterized by proximal muscle weakness, raised muscle enzymes, characteristic changes in electromyography and typical skin rash and biopsy findings. Dermatological features like Gottron's sign and papules are considered as pathognomonic for dermatomyositis. Panniculitis is one of the rare findings in dermatomyositis. Here, we report a case of dermatomyositis in 37 years old female who presented with only panniculitis and the diagnosis was delayed by more than a year.

## INTRODUCTION

Idiopathic inflammatory myositis are heterogeneous disorders which are characterized by weakness and inflammation of muscles.^1^ The most common subgroups of idiopathic inflammatory myositis in case of adults are dermatomyositis (DM), polymyositis (PM), and inclusion body myositis.^2^ Patients with pathognomonic skin rashes of juvenile DM or DM can be classified with the 2017 European League Against Rheumatism/American College of Rheumatology Classification Criteria for Adult and Juvenile Idiopathic Inflammatory Myopathies and their major subgroups without doing muscle biopsy. Muscle biopsy has been recommended for the patients without pathognomonic skin manifestations. Skin biopsy has been recommended for the dermatomyositis patients who have no muscle involvement.^3^ Gottron's papules and Gottron's signs are the pathognomic cutaneous manifestations seen in case of dermatomyositis. Other characteristic signs include heliotrope sign, nail fold changes, shawl sign, V sign, atrophic erythematous and scaly plaques of the scalp. Few cutaneous lesions which are compatible with dermatomyositis include poikiloderma, holster sign, periorbital edema and facial swelling. Bullous or necrotic lesions, cutaneous vasculitis and calcinosis cutis are rarely seen and flagellata, follicular hyperkeratosis, mucinosis, erythroderma, oral mucosa changes and panniculitis are even more unlikely feature.^4,5,6^ Here, we present a case of dermatomyositis, who presented initially only as panniculitis and the diagnosis was delayed by more than one year.

## CASE REPORT

**Figure 1:**
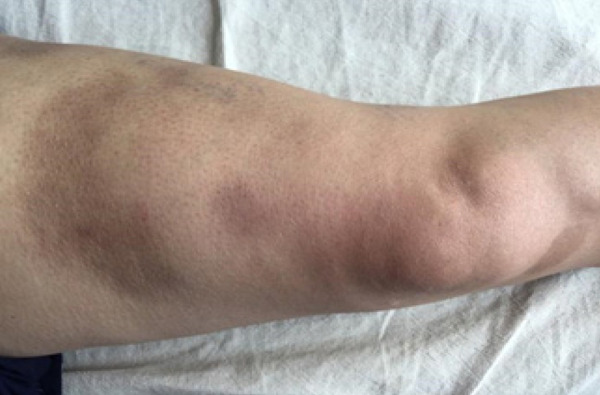
Multiple atrophic plaques over the right thigh.

**Figure 2:**
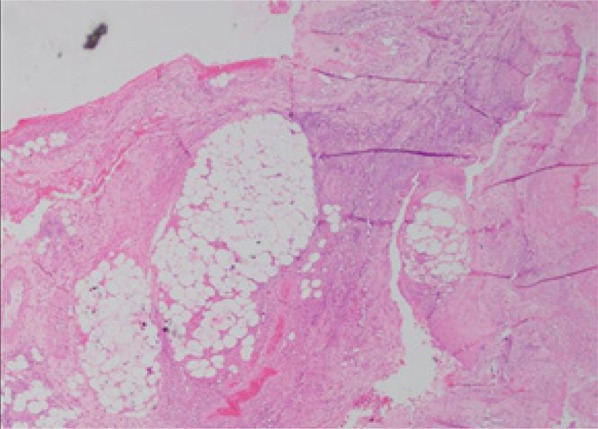
Biopsy from the atrophic plaque of thigh showing septal panniculitis.(hemoxylin and eosin stain 100x).

A 37 years old married female presented with a history of multiple ill-defined dusky erythematous tender nodules and plaques over bilateral thighs and arms for the last 15 months which were recurrent in nature. Most of the lesions healed with post-inflammatory hyperpigmentation with atrophy (Figure 1). The systematic examination was grossly unremarkable. A biopsy was done from the tender plaque which revealed septal panniculitis (Figure 2). Patient was started on oral prednisolone which had a dramatic response. On stopping the medications, flare-ups were noted which resolved with oral prednisolone so the patient took oral prednisolone on her own requirement.

One year back, the patient had developed redness of the face with photo-aggravation. Consultations were done and a provisional diagnosis of rosacea was made and treated accordingly without much improvement. Patient was also diagnosed with hypothyroidism and started on oral thyroxine but the facial lesion persisted.

Six months back, patient started experiencing generalized weakness, exertional dyspnea, myalgia, significant weight loss and multiple joints pain. Consultation with the rheumatologist was sought due to persistent myalgia and arthralgia. Physical examination revealed proximal muscle weakness. There was no synovitis. The examination also revealed diffuse erythema over the face predominantly over the bilateral periorbital region and upper back.

**Figure 3:**
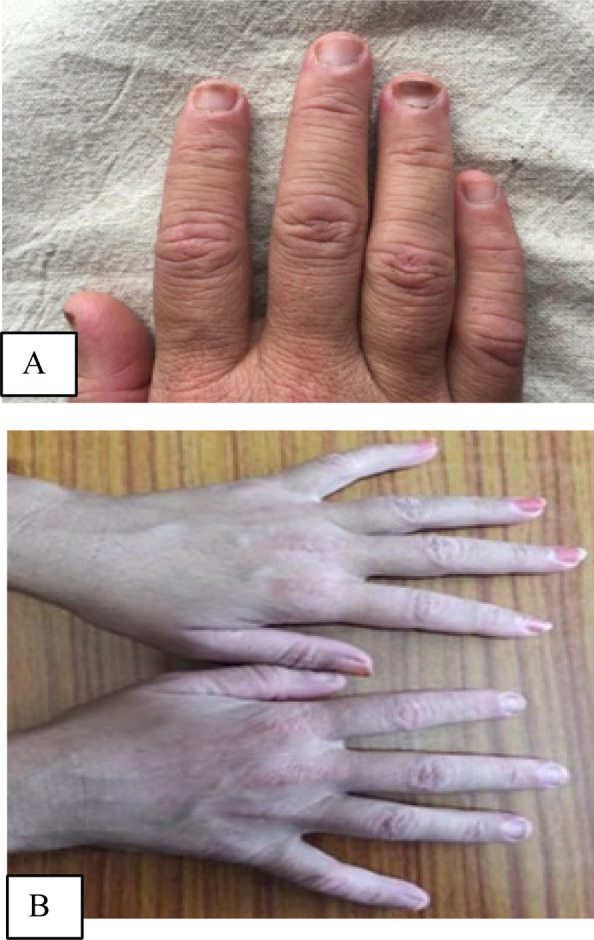
Figure showing prominent erythema over proximal nail folds (Figure 3A) and over the knuckles and the interphalangeal joints of most fingers (Figure 3B).

**Figure 4:**
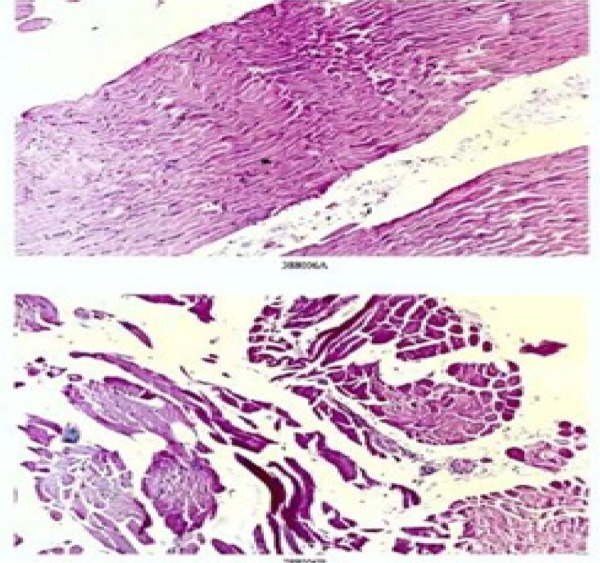
Figure showing mild distortion of fascicular architecture with moderate variation in size, perifascicular atrophy with internalization of nuclei, mild to moderate lymphoplasmacytic infiltrate around endomysial blood vessels and in endomysial and perimysial area. (Hematoxylin & Eosin stain 100X and 1000X).

There was mottled erythema with pigmentation over the V of the neck. There was a prominent erythema of proximal nail folds. Proximal nail folds and over the knuckles and the interphalangeal joints of most fingers (Figure 3A and 3B). Antinuclear antibody (ANA) test was done which was negative and muscle enzymes levels were all within normal limits. A strong suspicion of dermatomyositis due to the skin signs and proximal muscle weakness prompted an electromyogram (EMG) and muscle biopsy. EMG of the bilateral biceps muscle showed an increased insertional activity. A muscle biopsy performed from right quadriceps muscle showed mild distortion of fascicular architecture with moderate variation in size, perifascicular atrophy with internalization of nuclei, mild to moderate lymphoplasmacytic infiltrate around endomysial blood vessels and in endomysial and perimysial area (Figure 4). A high resolution computed tomography (HRCT) scan of chest showed patchy ground glass opacities. A computer tomography (CT) scan of abdomen and pelvis did not reveal any abnormalities.

Patient was classified as a definite case of idiopathic inflammatory myositis as per 2017 EULAR/ACR Classification Criteria for Adult and Juvenile Idiopathic Inflammatory Myopathies and their major subgroups with a score with muscle biopsy of 13.9. She was subsequently started on steroids and azathioprine with gradual improvement of arthritis, myalgia and subjective improvement in muscle strength. Patient is on regular follow-up.

## DISCUSSION

DM is a rare disease with a female preponderance. It has two peaks of incidence: first peak between 5 and 15 years of age and other between 40 and 60 years.^7^ Classically, DM presents with symmetric, proximal muscle weakness, and skin lesions that demonstrate interface dermatitis on histopathology. Clinically amyopathic DM is used to describe patients who have classic cutaneous manifestations for more than 6 months, without muscle weakness or elevation in muscle enzymes. Typical skin manifestations are heliotrope rash, Gottron's papules, Gottron's sign, the V-sign, and shawl sign. Less frequently observed manifestations include periungual telangiectasias, cuticular overgrowth, mechanic's hand, palmar papules overlying joint creases, poikiloderma, and calcinosis. The dermatological manifestations of DM can occur at any time without any relation to muscle weakness.^8^ There are few case reports on panniculitis as an initial presentation in our literature search.^9,10^ Our patient presented with rarely observed cutaneous manifestation in the form of panniculitis prior to the muscular and other common dermatological manifestations. She developed muscle weakness along with other cutaneous signs over a period of year. The HRCT of chest showed patchy ground glass opacities with mild interstitial septal thickening without honeycombing or nodularity. Though DM may be associated with malignancies the cancer screening in our patient was negative.

Our patient was subsequently managed with azathioprine and steroids with slow tapering. Patient gradually improved with improvement in muscle and cutaneous manifestation and is currently under regular follow-up. We reported this case because it presented with one of the rare cutaneous manifestations of DM. She later presented with muscular problems. Skin manifestation was misdiagnosed as rosacea, only to present with typical skin features in a year's time.

In conclusion, DM is a rare connective tissue disease which typically presents with proximal muscle weakness and skin signs. Many at times the presentations of disease may be atypical. There are certain rare presentations of DM including panniculitis. This case report brings out the need to think of connective tissue disease when we see rarer forms of presentations as well. Extensive evaluation might be required when the clinical suspicion is high but the initial evaluation is inconclusive.

## Consent:

**JNMA Case Report Consent Form** was signed by the patient and the original article is attached with the patient's chart.

## Conflict of Interest

**None.**
